# Radiographic assessment of femoral, acetabular and global offset following hip spacer implantation in staged total hip arthroplasty”

**DOI:** 10.1007/s00402-026-06384-3

**Published:** 2026-06-18

**Authors:** Luca Cavagnaro, Emilio Ferrari, Valentina Providenti, Vito Marciante, Giuliana Carrega, Matteo Formica

**Affiliations:** 1 Joint Replacement Unit/Bone Infection Unit (MIOS)–Ospedale Santa Maria di Misericordia, Via Martiri della Foce 40, 17031 Albeng, SV Italy; 2 Clinica Ortopedica-Policlinico San Martino, Largo Rosanna Benzi 10, 16132 Genoa, GE Italy

**Keywords:** Hip, Revision, PJI, Spacer, Two-stage, Complication

## Abstract

**Background:**

The number of total hip arthroplasties (THAs) performed worldwide is steadily increasing, and, consequently, the demand for revision procedures is expected to rise as well. two-stage revision procedure continues to be considered the gold standard in many clinical scenarios. During the first step, failure to restore femoral offset has been implicated as a contributor to instability and mechanical failure, although a clear consensus is still lacking.

**Objectives:**

The primary aim of this study is to evaluate the restoration of biomechanical parameters of the hip—specifically leg length discrepancy (LLD), femoral offset (FO), acetabular offset (AO), and global offset (GO)—following implantation of a specific type of articulating hip spacer during the first stage of a two-stage revision for PJI. A secondary objective is to assess the correlation between variations in offset parameters and the rate of interstage mechanical complications.

**Materials and methods:**

We retrospectively reviewed all patients undergoing staged revision with a specific articulating spacer between 2020 and 2022 at a single institution. Harris Hip Scores, Oxford Hip Scores, and Visual Analogue Scales for pain were obtained at different time points. Radiographic analysis included LLD, FO, AO and GO measurements on the affected (spacer and post-reimplantation) and the contralateral side. Complications were recorded during the interstage and post-reimplantation period.

**Results:**

Forty-seven patients (47 hips), were enrolled. The mean follow-up was 25.3 months. Clinical outcomes showed significant improvement from pre-operative visit to interim period and at the last follow-up (*p* < 0.01). No statistically significant differences for FO, AO and GO between contralateral and spacer side were observed. LLD before reimplantation was 10.1 ± 7.5 mm. GO increased of 4 ± 3.2 mm at final follow-up (*p* < 0.05) with a final LLD of 5.6 ± 4.9 mm. Six complications (12.8%) occurred during the interstage period: 4 spacer dislocations (8.5%) and 2 intraoperative femoral peri-spacer fractures (4.3%). Comparison between stable and dislocated spacers showed statistically significant differences for mean ΔFO (*p* = 0.02) and ΔGO (*p* = 0.04).

**Conclusion:**

The articulated spacer assessed in this study, especially when used in combination with a custom-made acetabular component, allowed approximation of the main biomechanical parameters, with small, non-significant differences compared to the contralateral side. Offset parameters reduction may be associated with spacer dislocation rate.

## Introduction

The number of total hip arthroplasties (THAs) performed worldwide is steadily increasing, and, consequently, the demand for revision procedures is expected to rise as well [1–4]. Periprosthetic joint infection (PJI) remains one of the leading causes of hip prosthesis failure, frequently requiring revision surgery [5]. While the one-stage revision approach has shown promising outcomes with good eradication rates in selected cases [6], the two-stage revision procedure continues to be considered the gold standard in many clinical scenarios due to its favorable long-term results [7]. Although the primary goal of revision THA in PJI is infection eradication, biomechanical preservation is a secondary but clinically relevant objective. In the first stage of the two-stage protocol, the use of articulating antibiotic-loaded spacers is the standard of care. These spacers offer several advantages: they help preserve joint function, reduce scar tissue formation—thereby facilitating second-stage reimplantation—and provide high local concentrations of antibiotics [8, 9]. Nevertheless, spacer use is not without complications. Reported mechanical complication rates, such as spacer dislocation, spacer fracture, perispacer fracture, and periprosthetic osteolysis, vary widely in the literature, ranging from 0 to 92% [10]. Several factors have been associated with an increased risk of spacer-related mechanical complications, including the presence of femoral or acetabular bone loss, inadequate femoral engagement, abductor muscle deficiency, and suboptimal spacer design or positioning [11–13]. Among these, restoration of key biomechanical parameters—particularly leg length discrepancy (LLD) and offset—has been increasingly recognized as a potentially modifiable risk factor [12, 14]. In particular, failure to restore femoral offset has been implicated as a contributor to instability and mechanical failure, although a clear consensus is still lacking [14, 15].

The primary aim of this study was to evaluate the restoration of hip biomechanical parameters—specifically leg length discrepancy (LLD), femoral offset (FO), acetabular offset (AO), and global offset (GO)—following implantation of a specific type of articulating hip spacer during the first stage of a two-stage revision for PJI. A secondary objective was to assess the correlation between variations in offset parameters and the rate of interstage mechanical complications.

## Materials and methods

A retrospective analysis was conducted on patients treated with staged revision for chronic hip PJI from January 2020 to December 2022. All data were prospectively collected in our Institutional Arthroplasty Register and retrospectively analyzed. The Institutional Review Board (IRB) approved this single-centre study (No. 007/2025). Written informed consent was obtained from all the included participants. All procedures were conducted according to the Declaration of Helsinki. All patients undergoing a two-stage hip revision with a specific articulating spacer (G21 SpaceFlex Hip) coupled with a handmade acetabular spacer during the study period were enrolled. Patients revised with different types of spacers or managed with an interim Girdlestone procedure were excluded. PJI diagnosis was made according to the 2018 International Consensus Meeting (ICM) criteria [16]. Main demographic variables (age, sex, diagnosis, affected side, body mass index [BMI], comorbidities, smoking status, previous surgical procedures) and surgical data (surgical time, surgical approach, acetabular and femoral bone loss, stem and acetabular spacer size, duration of the interstage period, the need of for a lateral window at the first stage, and final prosthetic implant type) were recorded.

Patients were classified according to the systemic host grade of the McPherson staging system [17]. Acetabular and femoral bone defects were classified radiographically before surgery and confirmed intraoperatively according to the classification of Paprosky et al. [18, 19].

### Clinical and radiographic evaluation

**Fig. 1:**
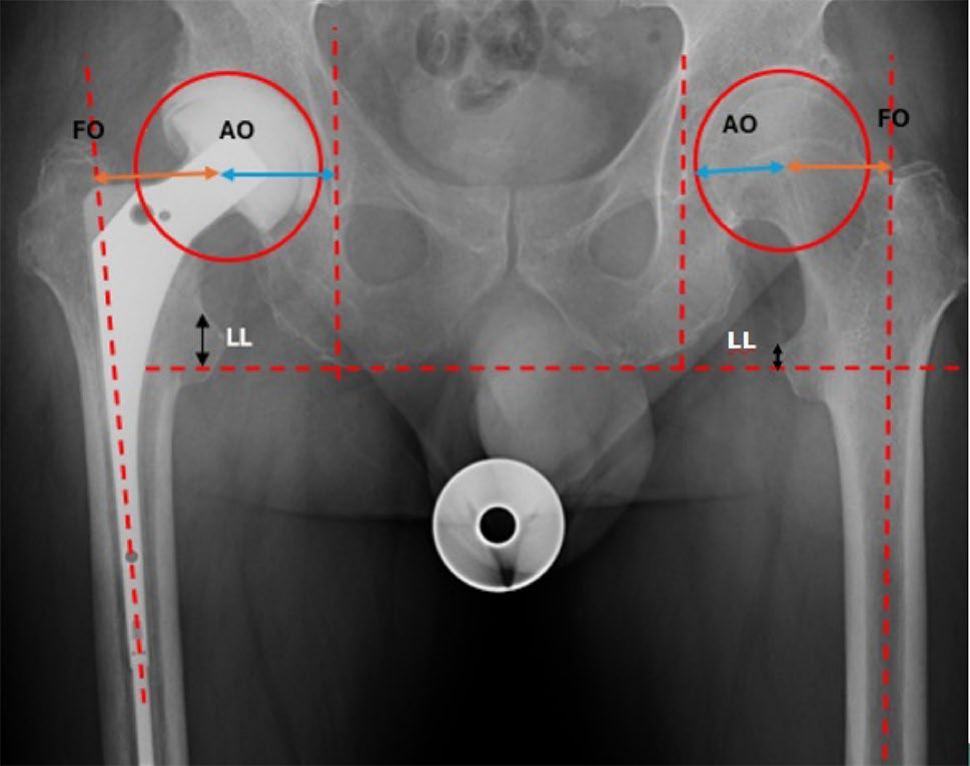
Measurements of the hip biomechanical radiographic parameters. *AO* acetabular offset, *FO* femoral offset, *LL* leg length

Clinical and radiographic evaluations were scheduled as follows: before the first stage; 6 weeks after the first stage; 1, 3, and 6 months after the second stage; and annually thereafter. Clinical assessment included physical examination, the Visual Analogue Scale (VAS), the Harris Hip Score (HHS), and the Oxford Hip Score (OHS), to evaluate subjective and objective hip function. Radiographic analysis included measurements of LLD, FO, AO, and GO on the affected side (spacer and post-reimplantation) and the contralateral side, as well as assessment of osseointegration, loosening, radiolucent lines, osteolysis, stem subsidence, malposition, and heterotopic ossification after final prosthetic reimplantation. Heterotopic ossifications were classified according to the Brooker classification [20]. Stem subsidence was defined as femoral stem distal migration greater than 2 mm observed on the latest AP radiograph compared with the immediate postoperative imaging [21]. Radiolucent lines and osteolytic areas were evaluated according to the Charnley-DeLee and Gruen classifications [22, 23] on the acetabular and femoral sides, respectively. Radiographic analysis was performed on standard anteroposterior (AP) radiographs obtained postoperatively with the patient in the supine position and both legs internally rotated by 15°. The X-ray beam was centered on the symphysis pubis. All measurements were performed twice at different time points on digital AP pelvic radiographs by the same author (VM), who was not involved in the index surgery. Recorded values represented the average of the two measurements. Radiological distances (LLD, AO, FO, and GO) were measured using Carestream Imageview software. FO was defined as the perpendicular distance from the center of rotation of the femoral head to the anatomical femoral axis [24]. AO was defined as the perpendicular distance from the center of rotation of the femoral head to a line passing through the medial edge of the ipsilateral teardrop, perpendicular to the line through the inferior margins of the ischial tuberosities [25]. GO was defined as the sum of FO and AO [26]. Limb length (LL) was defined as the distance between the medial apex of the ipsilateral lesser trochanter and the line passing through the inferior margins of the ischial tuberosities (Fig. 1). We measured AO, LL, and FO on both the operated and contralateral sides. Differences were defined as follows:

- ΔGO = spacer GO – contralateral GO
- ΔFO = spacer FO – contralateral FO
- ΔAO = spacer AO – contralateral AO
- LLD = spacer LL – contralateral LL

FO and AO were also assessed after reimplantation. Increases in offset measurements were expressed as positive values, whereas negative values indicated offset reduction. Complications were recorded during the interstage and post-reimplantation periods. Radiographs were assessed by two orthopaedic fellows (EF and VP).

### Surgical technique

**Fig. 2:**
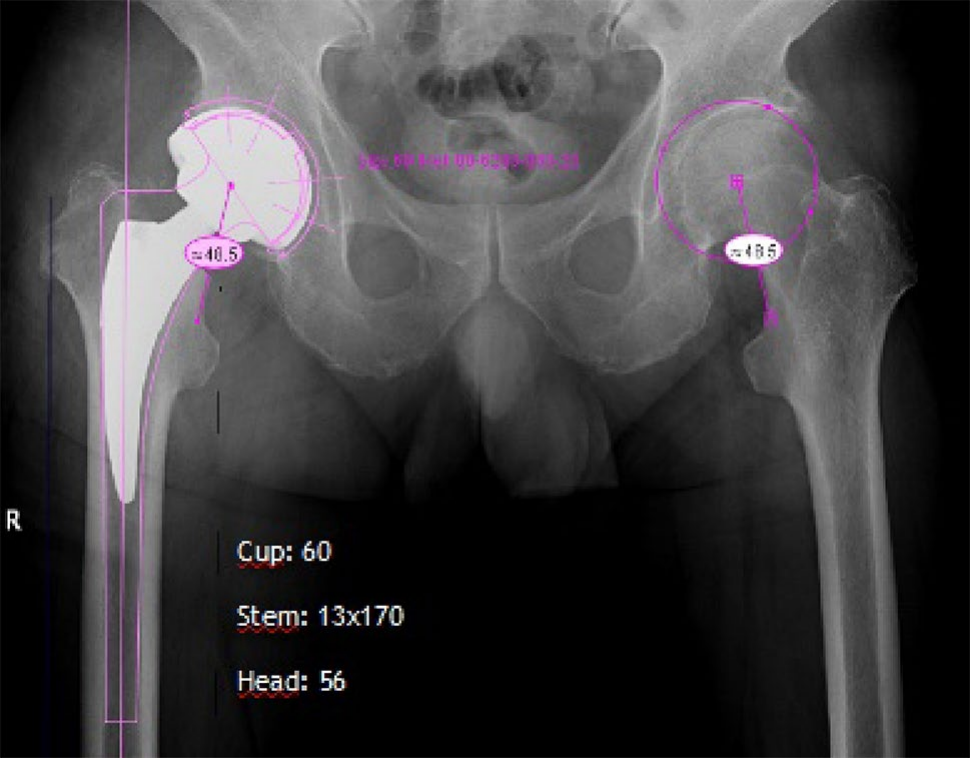
Preoperative planning of spacer implantation in right hip PJI

All patients underwent a two-stage procedure for hip PJI via a posterolateral approach. Preoperative digital templating (Fig. 2) with spacer templates was routinely performed to estimate size and head dimensions, though final sizing was determined intraoperatively after broaching the femoral canal and assessing bone loss.

Scar tissue, sinus tracts, and abscesses were excised. In the first stage, the prosthesis was explanted and a G21 Spaceflex hip spacer (G21 S.r.l., Modena, Italy) was implanted. Before antibiotic administration, at least five microbiological samples were collected while prosthetic components were sent for sonication.

**Fig. 3:**
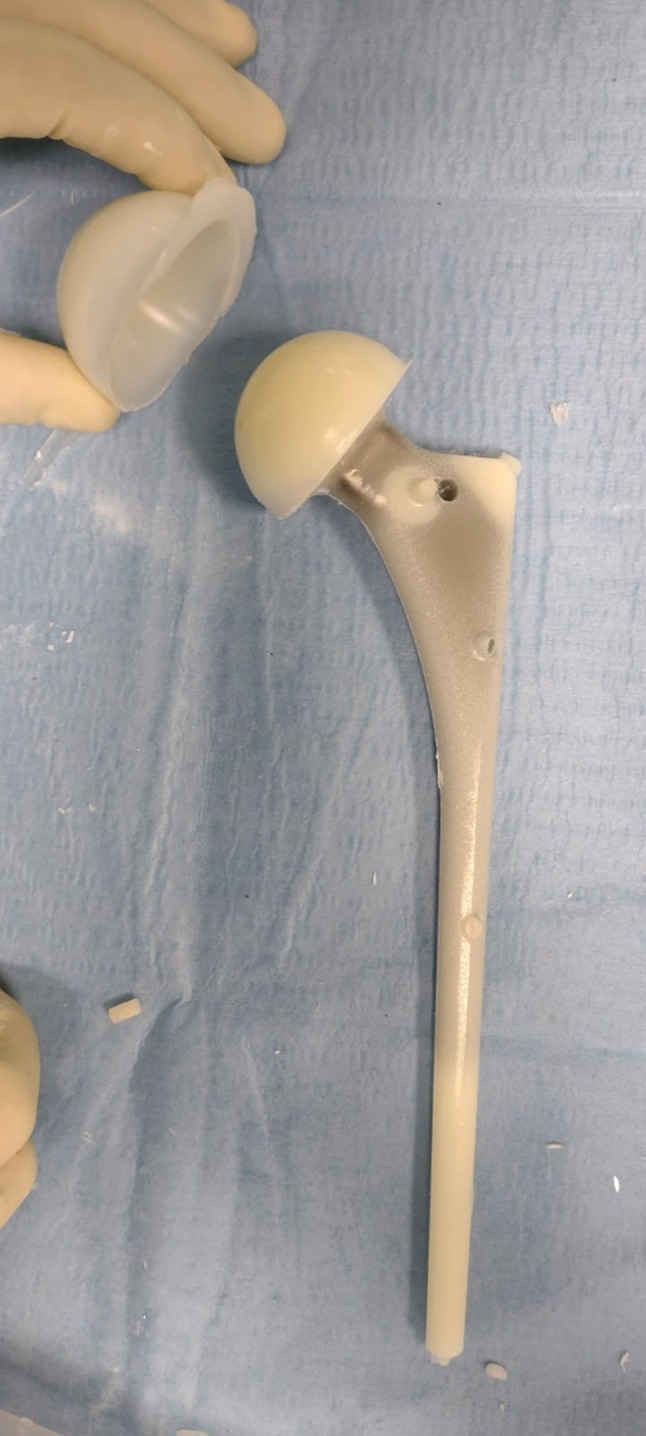
G21 Spaceflex hip spacer coupled with a custom-made acetabular spacer

For stem extraction, an extended lateral cortical window was performed if needed. After final intraoperative confirmation of spacer size, the G21 Spaceflex spacer was intraoperatively molded. It consists of a titanium core coated with gentamicin-loaded cement. Available options include three sizes of stem (10, 13 and 15) three possible lengths (140, 170, 210 mm) and 4 head sizes (48, 52, 56 and 60 mm). Acetabular spacers were handmade intraoperatively by molding a cement cup 2 mm larger than the spacer head [27] (Fig. 3). Low-viscosity G3A cement (G21 S.r.l., Modena, Italy) was used for molding the spacer, while PALACOS G^®^ gentamicin cement (Heraeus Medical GmbH, Hamburg, Germany) was used for fixing the devices. If needed, specific antibiotics were added to cement spacer according to preoperative microbiological data. Both acetabular and femoral spacers were loosely cemented to avoid excessive cement infiltration into cancellous bone. During reimplantation and after removal of the mobile antibiotic-loaded spacer, a new, accurate surgical debridement was performed. At least 5 intraoperative samples were collected, and spacer was sent for sonication. According to the size and shape of bone deficiency, the senior surgeon decided the best technique to address the bone defect and the final components.

### Postoperative course

After the first stage, partial weight-bearing with a walker or crutches was initiated on the second postoperative day after removal of the surgical drain and was continued throughout the entire interstage period. Standard venous thromboembolism prophylaxis with enoxaparin and compression stockings was prescribed for at least 45 days. In agreement with the infectious disease team, a 6-week course of targeted antibiotic therapy was administered (intravenously for 2 weeks, followed by oral administration for 4 weeks whenever possible, according to microbiological findings). After the second stage, physiotherapy was initiated with mobilization on the first postoperative day. Ambulation with partial weight-bearing was allowed on the second postoperative day. Standard venous thromboembolism prophylaxis with enoxaparin and compression stockings was prescribed for at least 35 days. An intravenous targeted antibiotic regimen was administered until intraoperative microbiological results became available and was subsequently continued if necessary.

### Statistical analysis

Continuous variables were reported as mean ± standard deviation (SD) and compared using paired or unpaired Student t test. Categorical variables were expressed as the number of cases and percentage and compared using chi-squared or Fisher’s exact tests. For all the analyzed data, a two-tailed, p value < 0.05 was considered statistically significant. For radiological parameters, interobserver reliability was evaluated with the Cohen’s kappa coefficient. We defined as re-operation any kind of surgery that involved the hip joint after the index procedure without removing the fixed prosthetic component. Conversely, revision was considered as any surgical procedure that required fixed component removal for any reason. Spacer revision was defined as any procedure performed on the affected hip during the interstage time. We defined septic recurrence as each new infection or positive culture at reimplantation with isolation of the original infecting organism. Statistical analysis was carried out with the XLSTAT and Excel statistical software.

## Results

 Overall, 47 patients (47 hips), who met all inclusion/exclusion criteria, were identified to be suitable to be enrolled in this study. All patients had a minimum follow-up of one year and none was lost during this time. The mean follow-up duration was 25.3 months. The mean age at surgery was 63.1 ± 11.4 years. Thirty patients were male (63.8%) and seventeen females (36.2%). The mean BMI was 26.2 ± 5.3. Excluding the index revision, patients had undergone an average of 2.3 ± 1.8 previous surgeries. Relevant comorbidities are summarized in Table 1. In all cases, chronic hip PJI was the indication for revision THA. Table 2 shows microbiological findings. The mean interstage period duration was 16.6 ± 11.1 weeks. Twelve (25.5%) patients required a femoral lateral window for stem extraction. Main surgical data along with femoral and acetabular bone defects distribution are potted in Table 3. According to spacer size, most of the included patient (55.3%) received a size 10 stem, 19 patients were implanted with a size 13 stem and only 2 had a size 15 spacer. Thirty-nine had a 48 mm spacer head; 52- and 56-mm heads were used in 4 patients each. Spacer length of 210 mm and 140 mm were employed in 29 (61.7%) and 17 (36.2%) of cases, respectively. Spacer features are showed in Table 4. 

**Table 1:** Demographic features

Variable	Value
Sex	
Male	30 (63.8%)
Female	17 (36.2%)
Mean age at explantation (years)	63.1 ± 11.4
Side	
Left (L)	18 (38.3%)
Right (R)	29 (61.7%)
BMI (kg/m²)	26.2 ± 5.3
Number of previous surgeries	2.3 ± 1.8
Diagnosis: PJI	47 (100%)
McPherson classification	
IIIA1	5 (10.6%)
IIIA2	10 (21.3%)
IIIA3	20 (42.6%)
IIIB1	–
IIIB2	2 (4.3%)
IIIB3	5 (10.6%)
IIIC1	–
IIIC2	–
IIIC3	5 (10.6%)

*BMI* body mass index, *PJI* periprosthetic joint infection

**Table 2:** Microbiological data

Pathogen type	N (%)
Polymicrobial	9 (19.1%)
MSSA	8 (17.0%)
MRSA	7 (14.9%)
MRSE	5 (10.6%)
CoNS	4 (8.5%)
Gram-negative bacteria	4 (8.5%)
MSSE	3 (6.4%)
Enterococci	3 (6.4%)
Culture negative	4 (8.5%)

MSSA methicillin sensitive staphylococcus aureus, *MRSA* Methicillin resistant staphylococcus aureus, *MRSE* methicillin resistant staphylococcus epidermidis, *CoNS* coagulase negative staphylococci, *MSSE* methicillin sensitive staphylococcus epidermidis

**Table 3:** Main surgical data

Variable	Category	N (%)
Acetabular defect (Paprosky)	Type 1	20 (42.6%)
	Type 2A	5 (10.6%)
	Type 2B	3 (6.4%)
	Type 2C	5 (10.6%)
	Type 3A	8 (17.0%)
	Type 3B	6 (12.8%)
Femoral defect (Paprosky)	Type 1	18 (38.3%)
	Type 2	15 (31.9%)
	Type 3A	8 (17.0%)
	Type 3B	6 (12.8%)
Acetabular spacer	Yes	47/47 (100%)
	No	0/47 (0%)
Mean interstage duration	–	16.6 ± 11.1 weeks
Lateral window	Yes	12 (25.5%)
	No	35 (74.5%)
Re-revision	Yes	8 (17.0%)
	No	39 (83.0%)

**Table 4:** Spacer characteristics

Spacer parameter	Size	N (%)
Hip spacer size	10	26 (55.3%)
	13	19 (40.4%)
	15	2 (4.3%)
Spacer head diameter	48	39 (83.0%)
	52	4 (8.5%)
	56	4 (8.5%)
Spacer stem length	140	17 (36.2%)
	170	1 (2.1%)
	210	29 (61.7%)

### Clinical and radiological analysis

 The mean HHS and OHS improved significantly from 29.7 ± 9.3 and 18.4 ± 10.6 pre-operatively to 72.6 ± 7.8 and 31.6 ± 3.3 before reimplantation and, 84.1 ± 8.9 and 39.4 ± 4.0, respectively, at the last follow-up (*p* < 0.01). On average, VAS decreased from 8.4 ± 2.1 pre-operatively to 1.1 ± 1.2 at the last evaluation (*p* < 0.01). Seven patients walked with crutches and 3 had mild limping at final follow up. Mean differences for FO, AO and GO between contralateral and spacer side were 0.4 ± 9.9, − 2.7 ± 6.0 and −2.3 ± 7.7 mm, respectively with no statistically significant differences for the observed values (Table 5). LLD before reimplantation was 10.1 ± 7.5 mm. We observed a significantly mean increase of GO of 4 ± 3.2 mm at final follow-up (*p* < 0.05) with a final LLD of 5.6 ± 4.9 mm. No cases of migration, loosening, or stem subsidence were observed at the last follow-up radiographic analysis. Two cases (4.3%) showed incomplete and not progressive < 2 mm radiolucent lines on Gruen zone 1. Heterotopic ossifications (2 Brooker type 2 and 1 Brooker type 3) were observed in 3 patients (6.4%). No dislocations were reported during the post-reimplantation period. For radiological parameters, very good (≥ 90%) Cohen’s kappa inter-rater agreement was found.

**Table 5:** Offset data comparison between spacer and controlateral hip

Offset parameter	Spacer (Mean ± SD)	Contralateral hip (Mean ± SD)	Δ (Difference)	p-value
Femoral Offset (mm)	48.4 ± 8.6	48.0 ± 9.9	+0.4 ± 9.9	0.93
Acetabular Offset (mm)	35.1 ± 6.0	37.8 ± 6.2	−2.7 ± 6.0	0.39
Global Offset (mm)	85.5 ± 9.6	87.8 ± 12.5	−2.3 ± 7.7	0.45

*SD* standard deviation

### Complications

 Six complications (12.8%) occurred during the interstage period (Table 6): 4 spacer dislocations (8.5%) and 2 intraoperative femoral peri-spacer fractures (4.3%) during the first stage. No cases of spacer fracture, postoperative peri-spacer fracture or spacer revision for persistent infection were reported. Two dislocations were managed with spacer revision, 1 underwent a closed reduction with no subsequent relapse and 1 patient was managed with final prothesis reimplantation. Comparison between stable and dislocated spacers showed statistically significant differences for mean ΔFO (*p* = 0.02., 95% CI 12.5–26.5) and ΔGO (*p* = 0.04, 95% CI 13.0–26.2), no significant differences were detected for other commonly involved parameters affecting dislocation (Table 7). Four (8.5%) of the included patients reported complications, including 1 septic recurrence (2.1%), 1 new prosthetic infection (2.1%) managed with a repeated two stage procedure, and 2 positive intra-operative microbiological cultures managed with specific suppressive therapy for three months with good outcome. The overall final implant survival rate was 95.7% at final follow-up.

**Table 6:** Interstage complications

Complication type	N	%
Dislocation	4	8.5%
Peri-spacer fracture	2	4.3%
Spacer fracture	0	0.0%
Infection recurrence	0	0.0%

**Table 7:** Comparison between dislocators and non-dislocators

Variable		Non dislocators	Dislocators	p-value
Massive acetabular defects (n°)		13	1	1.0
Massive femoral defects (n°)		13	0	0.56
Spacer head size	48	36	3	0.53
	52	3	1	0.30
	56	4	0	1.0
Spacer length	140	16	1	1.0
	170	1	0	1.0
	210	26	3	1.0
Abductor mechanism disruption (n°)		5	1	0.43
Δ AO (mean+SD)		+ 0.9 ± 7.0	- 1.8 ± 8.0	0.40
Δ FO (mean+SD)		+ 2.0 ± 8.8	- 17.5 ± 6.6	**0.02**
Δ GO (mean+SD)		+ 0.3 ± 9.7	- 19.3 ± 6.0	**0.04**
Δ LLD (mean+SD)		- 9.6 ± 3.7	- 10.4 ± 1.2	0.34

Statistically significant values are highlighted in bold

## Discussion

The primary objective of this study was to assess the ability of the G21 Spaceflex articulating spacer to restore key hip biomechanical parameters—FO, AO and GO, as well as leg length—in patients undergoing staged revision for periprosthetic hip infection. The findings demonstrate that, in most cases, the spacer approximated native biomechanical parameters, although small differences were observed and their clinical relevance remains uncertain, thereby offering a reliable interim reconstruction during the first stage of revision. The articulating spacer used in this study, combined with a custom-made acetabular component and systematic preoperative templating, was designed to provide temporary joint articulation and maintain soft-tissue tension during the interstage period with optimal infection control. This approach may help limit limb shortening and offset reduction, which are known contributors to instability and mechanical complications during the spacer phase. A notable observation emerged when comparing patients who sustained spacer dislocation with those who did not. Patients with dislocation exhibited a statistically significant reduction in femoral and global offset, whereas no significant differences were detected in other parameters commonly associated with instability. These findings should be interpreted with extreme caution due to the very low number of events, and no reliable inference regarding predictors of dislocation can be made. Rather, these results are hypothesis-generating and support the possibility that inadequate restoration of offset may contribute to soft-tissue imbalance and reduced joint stability, ultimately increasing the risk of spacer dislocation. This exploratory observation aligns with prior reports emphasizing the central role of offset and soft-tissue tension in maintaining stability during staged revision procedures [28, 29]. The present findings are consistent with the literature, which underscores the challenges of achieving optimal biomechanics with prefabricated spacers [13, 15]. Most commercially available spacers provide a fixed neck–shaft angle and a single offset option, limiting their adaptability to the broad anatomical variability encountered in revision settings [11]. These inherent constraints have been previously associated with suboptimal joint biomechanics and instability. Despite these limitations, the spacer used in this study—particularly when combined with a custom-made acetabular component— achieved values close to the contralateral side in the majority of patients, although the clinical relevance of these small differences remains uncertain. Although our study does not demonstrate a direct clinical impact of offset restoration, this suggests that the combined use of modular or custom acetabular elements may mitigate some of the intrinsic limitations of standard femoral spacers and improve biomechanical reconstruction [27, 28]. Mechanical complications of hip spacers, particularly dislocation, remain a major concern, with reported rates ranging from 0% to 92.3% in the literature [10]. Risk factors for spacer dislocation include poor patient compliance, inadequate spacer femoral engagement, undersized spacer head, large acetabular bone defects, decreased leg length and muscular insufficiency [12].

Spacer geometry is also critical: Leunig et al. found that higher neck-to-head ratios increased dislocation risk, while insufficient intramedullary anchorage (< 22 ± 33 mm) was associated with failure [29]. Jones at al retrospectively reviewed 185 patients treated for hip PJI with antibiotic cement spacers between 2004 and 2014. Dislocation occurred in 9% and was significantly associated with reduced femoral offset of > 5 mm and increased bone loss. The authors concluded that spacer design, acetabular and femoral bone loss, and offset restoration were significantly associated with perioperative complications. Nonetheless, Molinas et al. [30] reported that lateral femoral offset (LFO) and modified vertical femoral offset (MVFO) did not modify dislocation rate. Although the mean LFO reduction on the spacer side was 8 mm, the same parameter for MVFO was only 1.2 mm. This minimal offset reduction explained the non-statistical correlation between offset reduction and dislocation rate. In order to improve spacer geometry, several techniques have been proposed with acceptable biomechanical and functional results. PROSTALAC (prosthesis of antibiotic-loaded acrylic cement, DePuy Synthes, Warsaw, IN, USA) was initially developed for septic hip revision and consists of a metal femoral stem and a polyethylene acetabular liner [31]. In a 10- to 15-year follow-up study, 99 PJI patients using the PROSTALAC hip spacer attained an 89% long-term treatment success rate [32] however, the availability of PROSTALAC is limited, and it is currently not approved for use in many countries. Custom-made articulating spacers (CUMARS) system the Exeter Universal Femoral stem (Stryker, Mahwah, NJ, USA) and a polyethylene acetabular liner [33]. It is comparable to PROSTALAC, but its components are more common and readily available. Recently, CUMARS was reported to be associated with good interstage functionality, easier removal, and excellent infection control [34] but polyethylene can act as a substrate for infection relapse with a possible increase of failures. Moreover, this procedure is burdened by considerable costs.

The post-reimplantation biomechanical analysis showed values close to the contralateral side of offset and leg length parameters with no dislocations during the follow-up period. This underlines a proper restoration of hip anatomy when compared to the preoperative contralateral side. This goal should be achieved only with proper preoperative planning and intraoperative accurate component positioning considering the first stage not only a debridement phase but the first step of hip reconstruction. From the clinical point of view, main objective and subjective scores improved significantly form the preoperative assessment but no clear association between functional outcomes (HHS, OHS, VAS) and radiographic parameters or complications was demonstrated, and that these results should be interpreted descriptively. This study has several limitations. Its retrospective design, although based on prospectively collected data, may introduce selection bias and prevents definitive causal inference. A second limitation is that, although 47 patients were included, only 4 dislocation were recorded, limiting the possibility to make clear estimations and adjusted analysis according to confounding variables but depicting only suggestive trends. Furthermore, the lack of a control group limits comparative interpretation showing potential advantage*s* rather than proven superiority of the described technique. The study design does not allow any conclusions regarding the relative performance of this spacer compared to alternative techniques. The limited follow-up does not allow evaluation of long-term mechanical or functional outcomes. Absolute values of some radiographic differences are small and should be interpreted with caution and primarily serve as a descriptive assessment of spacer geometry rather than functional determinants. The cohort shows substantial variability, in acetabular, femoral defects and the use of a lateral window. These factors may significantly affect stability, offset restoration, and complication risk. Finally, we measured the biomechanical parameters on the AP x-ray while some authors argued that CT-scans are more accurate [35]. CT scans were not routinely used to control patients in the interim period. In order to minimize measurement errors, we standardize x-ray protocol and all measurements were taken twice at different time points. Nevertheless, the present work also has notable strengths, including a consecutive and homogeneous patient series, a uniform surgical approach performed by a single surgeon, and the absence of dropouts, all of which enhance the reliability and consistency of the data.

## Conclusions

The articulated spacer assessed in this study, especially when used in combination with a custom-made acetabular component, allowed approximation of the main biomechanical parameters of the hip, although the clinical relevance of the small observed differences remains uncertain, in most patients undergoing two-stage revision for infection. It should be considered a safe and effective interim solution for staged hip revision providing good clinical and functional outcomes in the interstage period. Offset reduction may be associated with spacer dislocation rate. Although these findings are hypothesis-generating only and cannot be used to guide clinical decision-making, the possible association between insufficient offset restoration and spacer dislocation highlights the importance of meticulous preoperative planning and accurate component positioning to reduce mechanical complications during the interim stage. These findings may contribute to improving the management strategies for periprosthetic hip infection and optimizing patient outcomes.

## Data Availability

No datasets were generated or analysed during the current study.
